# The Application of Organic Matter Temporarily Shifts Carrot Prokaryotic Communities in the Endosphere but Not in the Rhizosphere

**DOI:** 10.3390/microorganisms11102377

**Published:** 2023-09-23

**Authors:** Irem Bagci, Kazuki Suzuki, Rasit Asiloglu, Naoki Harada

**Affiliations:** 1 Graduate School of Science and Technology, Niigata University, Niigata 950-2181, Japan; irembagci98@gmail.com; 2 Institute of Science and Technology, Niigata University, Niigata 950-2181, Japan; suzukik@agr.niigata-u.ac.jp (K.S.); asiloglu@agr.niigata-u.ac.jp (R.A.)

**Keywords:** 16S rRNA gene metabarcoding, carrot, endosphere, organic matter application, prokaryotic community composition, rhizosphere

## Abstract

Endophytic prokaryotes, bacteria, and archaea, are important microorganisms that benefit host plants by promoting plant growth and reducing stress. The objective of this study was to evaluate temporal shifts in the root endophytic prokaryotic communities associated with carrots (*Daucus carota* subsp. *sativus*) and the effect of organic matter application on them. Carrots were grown in a planter under five fertilizer treatments (weed compost, bark compost, cattle manure, chemical fertilizer, and no-fertilizer control) and the compositions of rhizosphere and root endosphere prokaryotic communities were determined via amplicon sequencing analysis targeting the 16S rRNA gene at 60 and 108 days after sowing. The results showed that the rhizosphere prokaryotic community compositions were stable despite different sampling times and fertilizer treatments; however, a greater temporal shift and an effect of the type of organic matter applied were observed in the endosphere prokaryotic communities. The differences in treatments resulted in significant differences in the abundance and Faith pyrogenetic diversity of the endosphere prokaryotic community. Genera, such as *Burkholderia*, *Sphingomonas*, and *Rhodanobacter*, that exhibit plant-growth-promoting and biocontrol activities, were detected regardless of the treatments, suggesting that they may play an important ecological role as the core endophytes in carrot roots.

## 1. Introduction

There has been a growing interest in research on the ecology of soil microorganisms and plant–microbe interactions in order to improve crop growth and yields or to maintain natural ecosystems. Terrestrial plants usually coexist with the soil microbiome, a very diverse microbial community including soil bacteria, fungi, protists, archaea, and viruses [1,2]. A thin layer of soil around the roots is called a rhizosphere and is constantly under the influence of plants. Plants secrete organic compounds from the roots, making them a “hot spot” for a variety of microbial activities. The rhizobacterial community is therefore one of the most complex ecosystems and forms a community which is distinct from those in the bulk soils, which serve as the natural habitat for soil-specific microbes. Some of the rhizobacteria enter the roots of the plant and are called endophytic bacteria. In this process, plant roots play an important role as gatekeepers for screening bacteria in the rhizosphere [3]. Consequently, the root endosphere, rhizosphere, and bulk soil all have different microbial communities [4].

Recently, several excellent reviews have been written on the role of endophytes in promoting plant growth and reducing plant stress [5,6,7,8]. Endophytic bacteria have been isolated and characterized from a variety of plant hosts. These include annual and perennial plants, such as common vegetables, grasses, wild plants, and plants growing in extreme environments [9,10,11]. Endophytic bacteria not only support the growth of the host plant but also help the host tolerate stress conditions and produce biological effects on other competing plant species. Endophytes then enable the host to better survive against biotic and abiotic changes in the environment. Many bacterial and fungal endophytes have been evaluated for the ability to increase the availability of nitrogen (N), phosphorus (P), and mineral nutrients such as potassium (K) and iron (Fe) to plants [12,13]. Other studies have also shown that endophytes specifically increase the elemental composition of N and P in plant roots and shoots [14,15,16].

In addition, a nutrient acquisition process called the “rhizophagy cycle” has recently been postulated [17]. According to this theory, microbes initially spend part of their life as free-living forms in the soil, accumulating nutrients. Subsequently, they colonize the rhizoplane within the exudate zone adjacent to the root meristem and then enter root tip meristem cells. Within the root periplasmic spaces, the plants transform microbes into wall-less protoplasts and extract their nutrients. The surviving microbes facilitate the elongation of root hairs and escape from their tips into the surrounding soil.

Carrot (*Daucus carota* subsp. *sativus*) is one of the ten most economically important vegetable crops worldwide in terms of production area and market value [18], and its production area has been increasing in recent years. Carrot is also popular in Japan, and is grown on 16,800 ha with an annual production of 585,900 t [19]. Carrot contains a number of health-promoting functional components such as carotenes, vitamins B2 and C, folic acid, carotenoids, and dietary fiber, and is also known as an important source of naturally occurring antioxidants with potential anti-cancer properties [20]. Consumption of carrots and their derivatives is steadily increasing.

Endophytic bacterial colonization in the roots of carrots was first reported by Surette et al. [21]. They isolated 360 isolates and identified 28 genera including *Pseudomonas*, *Staphylococcus*, and *Agrobacterium* as the most common bacterial endophytes. More recently, endophytic microorganisms were examined in the context of disease management (*Alternaria dauci*) in carrot production, and 28 unique isolates that belonged to at least 13 genera were isolated from carrot roots grown in a long-term trial comparing organic and conventional cultivation [22]. They belonged to Proteobacteria, Firmicutes, and Bacteriodes, which were the dominant phyla [22]. Only a few studies have comprehensively investigated the endophytic microbial community of carrot roots. Fungal communities associated with carrot roots have been reported by Abdelrazek et al. [23], but, to the best of our knowledge, there are no reports available on the endophytic bacterial communities.

As described, numerous studies have reported endophytic microbes that can enhance crop growth, but knowledge of carrot endophytes is limited. The formation of endophytic microbial communities within the roots is thought to be influenced by the crop farming environment, weather, and human activity; however, many unknowns remain. The objective of this study was to evaluate, by means of a planter experiment, temporal shifts in the prokaryotic communities, namely bacterial and archaeal communities, in the rhizosphere and endosphere of carrot roots, one of the most commonly consumed vegetables worldwide, and the impact on them of organic matter application.

## 2. Materials and Methods

### 2.1. Experimental Setup

The soil sample for this study was collected in September 2021 from a fallow upland field on the Niigata University, Ikarashi Campus (N37.87140, E138.94561). The soil type is sandy regosol, according to the comprehensive soil classification system of Japan. The soil was used after a sieving process (4.75 mm mesh). The amount of fertilizer to be applied was calculated as shown in Table 1 and Table S1, in accordance with the local fertilization standard. In this study, the P_2_O_5_ application dose was covered by each organic fertilizer, and the deficient N and K_2_O were supplemented using chemical fertilizers. This was because it is unrealistic to use only organic fertilizers to provide the amount of N required for carrot cultivation.

Before transferring the sieved soil to the planters, the soil was mixed with the fertilizers as specified in Table 2. This study used fifteen planters consisting of three replications from four fertilizer treatments (weed compost [WC], bark compost [BC], cattle manure [CM], and chemical fertilizer [CF]) and one control without fertilizer (CT). The soil and fertilizer mixture was transferred to a planter measuring 620 × 280 × 230 mm (length × width × depth).

**Figure 1:**
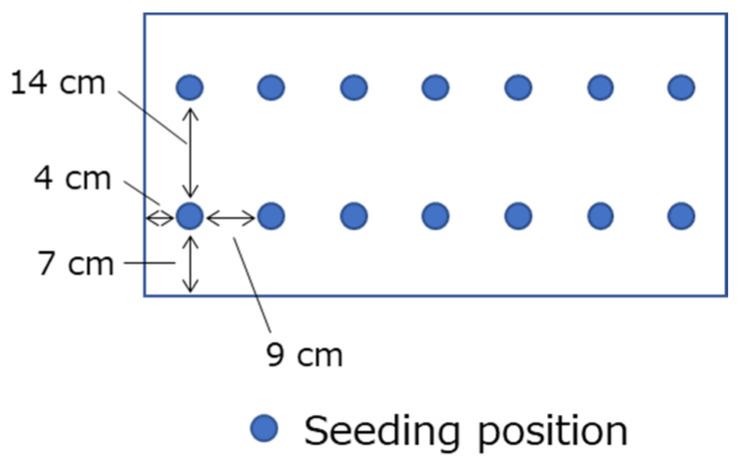
Seeding positions in a planter.

Then, carrot seeds (cultivar Baby carrot, Sakata Seed Co., Yokohama, Japan) were sown on 10 September 2021 (defined as 0 DAS [days after sowing]). The seeding positions in a planter are shown in Figure 1. The initial planter positions were randomly determined under a randomized block design and the planter position was changed monthly within each block. From sowing to harvest, irrigation was carried out as needed with tap water.

### 2.2. Sampling Collection and Preparation

During the production season, sampling was performed twice (60 and 108 DAS). At each sampling, three different soil samples (bulk soil and rhizosphere soil) and three carrot crops were collected from each planter. First, the carrot samples were cleaned several times with tap water before being washed in an ultrasonic water bath; the roots were separated, and then, shoot and taproot biomass (dry weight) were determined. Separately, fine roots were collected and 0.04 g of them was crushed after being frozen with liquid N_2_ and stored at −80 °C in a 2 mL tube for the microbiological analysis described in Section 2.4. Similarly, the rhizosphere soil samples were separated into 2 mL tubes and stored at −80 °C for microbiological analysis. Bulk soil samples were used for available P, pH, and EC analysis after being air-dried. For TC, TN, and CN analysis, the soils dried for 1 day at 105 °C were used. These dried soil samples were stored in a cold room until analysis.

### 2.3. Soil Physicochemical Analysis

Soil pH was measured with a pH meter (LAQUA, HORIBA, Kyoto, Japan) equipped with a composite glass electrode at a soil/water ratio of 1:2.5. Soil electronic conductivity (EC) was measured by inserting the glass electrode of the EC meter (TOA Electronics, Tokyo, Japan) into the 1:2.5 suspension. Total carbon and nitrogen contents (TC and TN, respectively) of the dried soil sample were measured using a CN Coder (Yanaco Technical Science, Kyoto, Japan), and the C/N ratio was calculated. Available phosphate (P) was extracted via the Truog method and the phosphate concentration in the extract was determined via the molybdenum blue method. The absorbance at 710 nm was analyzed using a spectrophotometer (UV-160A, Shimadzu, Kyoto, Japan).

### 2.4. DNA Extraction, Amplification, and Sequencing

Using an ISOPLANT kit (Nippon Gene, Tokyo, Japan), DNA was extracted from 0.2 g of homogenized root tissues. The ISOIL for Beads Beating kit (Nippon Gene) was used to extract DNA from soil samples (0.5 g). The universal primer pair 515F/806R [24] with overhang adapter sequences for the Nextera XT index primers was used for the initial amplification of the partial 16S rRNA genes of prokaryotes (Illumina, San Diego, CA, USA). The total volume of the PCR solution was 25 µL, which included 1 µL of template DNA, 1 µL of each of the primers (final concentration: 0.2 µM), 2.5 µL 10 × ExTaq buffer, 2.0 µL dNTPs, and 0.125 µL Ex Taq (Takara Bio, Kusatsu, Japan). For the root DNA samples, the PCR condition was set as follows: an initial 2 min denaturation step at 94 °C was followed by 35 cycles of denaturation at 94 °C for 30 s, annealing at 57 °C for 30 s, and elongation at 72 °C for 20 s, followed by a final extension at 72 °C for 5 min. For the soil DNA samples, PCR condition was set as follows: an initial 2 min denaturation step at 94 °C was followed by 25 cycles of 94 °C for 30 s, 57 °C for 30 s, and 72 °C for 20 s, followed by a final 5 min elongation step at 72 °C for all soil samples. The PCR products were then purified using Agencourt AMPure XP (Beckman Coulter, Brea, CA, USA) according to the manufacturer’s instructions. The 2nd PCR was performed with Nextera XT index kit v2 (Illumina, San Diego, CA, USA) under the following conditions: a 3 min denaturation step at 95 °C; 10 cycles of 94 °C for 30 s, 55 °C for 30 s, and 72 °C for 30 s; and a final 5 min elongation step at 72 °C. The 2nd PCR amplicons were purified as described above. The DNA concentration in the sample was quantified using QuantiFluor (Promega, Madison, WI, USA). Finally, an equimolar concentration solution of the purified amplicons was prepared. Paired-end sequencing was performed at a read length of 2 × 300 bp on the Illumina MiSeq platform with the MiSeq reagent kit v3 (Illumina).

### 2.5. Bioinformatics and Statistical Analysis

After MiSeq sequencing, the raw FASTQ files were processed using the QIIME2 pipeline (version 2018.8; http://qiime2.org/; accessed on 25 August 2023) [25]. For data processing (data pairing, primer trimming, quality filtering, chimera removal, and generating amplicon sequence variants [ASVs]), Illumina amplicon sequence calling was performed using the DADA2 algorithm [26]. Bacterial and archaeal identification of each sequence was performed based on the SILVA 138 database. ASVs derived from eukaryotes, mitochondria, and chloroplasts were excluded from subsequent analyses. The rhizosphere samples were resampled at 10,000 sequencing depths, while the endosphere samples were resampled at 4000 sequencing depths for alpha and beta diversity analyses.

To investigate alpha diversities of the soil bacterial and archaeal communities, richness (the number of ASVs), Shannon’s diversity index, and Faith’s phylogenetic diversity were calculated. The two-way ANOVA was used to examine the differences in the prokaryotic alpha diversities (Shannon diversity and Faith phylogenetic diversity) and richness as a function of the applied fertilizer type and carrot growth period. The beta diversity of the prokaryotic communities was analyzed through principal coordinate analysis (PCoA) and permutational analysis of variance (PERMANOVA) based on the weighted UniFrac distance with 9999 permutations. Relationships of environmental and carrot growth factors to the prokaryotic beta diversities were fitted onto the ordination using the envfit function of “vegan” 2.6-4 package of R version 4.2.3 [27]. Using the online platform InteractiveVenn [28], Venn diagrams were generated to illustrate the shared and unique bacterial taxa at the genus level among the treatments at 60 and 108 DAS. Prokaryotic genera that were found in two or more replicants and showed a relative abundance of 0.5% or more were used to generate Venn diagrams.

All the data were subjected to statistical analyses. Tukey’s test (*p* < 0.05) was performed to compare the means for each variable using the R “vegan” 2.6-4 package [27]. Paired *t*-test was applied to detect significant differences between both sampling dates.

## 3. Results and Discussion

### 3.1. Soil Physicochemical Properties

Firstly, the effect of the different fertilizer treatments on the physicochemical properties of the bulk soil, including the pH, EC, available P, TC, and TN, and C/N ratio, were examined. The results are shown in Table 3. The organic matter content of the soil did not change TC and TN contents even for CM, BC, and WC treatments, possibly due to a single application of a normal dose of organic matters in this experiment.

The soil pH ranged from 4.18 to 4.40 at 60 DAS and slightly decreased to 4.11–4.21 at 180 DAS. A significant decrease between 60 and 108 DAS was observed only in the BC sample. The CF and CM samples had significantly lower pH values than the control at 60 and 108 DAS. The EC varied between 0.54 and 1.30 mS/cm at 60 DAS and between 0.21 and 0.48 mS/cm at 108 DAS. No significant difference between the treatments was found on both sampling dates. All fertilizer treatments except CF significantly decreased soil EC from 60 DAS to 108 DAS.

The soil’s available P contents were between 0.22 and 0.30 g-P_2_O_5_/kg-ds at 60 DAS and between 0.40 and 0.48 g-P_2_O_5_/kg-ds at 180 DAS, with a significant 56–90% increase at 108 DAS compared with 60 DAS. The WC treatment showed significantly lower available P content compared with the CT at 60 DAS. The CF, BC, and WC treatments showed significantly lower available P in the soil when compared with the CT at 180 DAS. The CT showed the largest value on both sampling dates, likely due to low P absorption by the carrots due to their poor growth, as described in Section 3.2.

The TC content, TN content, and C/N ratio showed no significant difference between the CT and other fertilizer treatments, indicating that the organic and chemical fertilizer treatments had little effect on soil organic matter content in this study.

### 3.2. The Effect of Organic and Chemical Fertilizers on Carrot Growth

The effect of organic and chemical fertilizers on carrot growth was examined by measuring shoot and taproot biomass and the results are shown in Table 4. The best shoot biomass was found in the BC sample, followed by CM treatment for both 60 and 108 DAS. However, no significant effect of the treatments was observed, due to the wide variations. Taproot biomass ranged from 0.18 to 1.86 g at 60 DAS and from 2.50 to 7.66 g at 108 DAS. On both dates, the values in the BC sample were the largest. On the other hand, the smallest taproot biomass was found in the CT and WC treatment at 60 and 108 DAS, respectively. The CF and CM samples showed values between these.

In this experiment, the same amount of N, P_2_O_5,_ and K_2_O was applied, except for the CT. We had expected that the CF treatment, where the whole nutrients were provided as inorganic substances, would show the best carrot growth, but in fact, BC treatment showed the largest shoot and taproot biomass. Thus, this experiment confirmed that the application of compost, especially bark compost, has a positive effect on the growth of carrots. Compost is known to improve plant growth in several ways, including increasing nutrient content, improving soil moisture retention, and reducing the impact of pests [29]; however, the shoot and taproot biomass at 108 DAS increased by 24% and 88%, respectively, in the BC treatment compared with the CF treatment in this study. This suggests that some growth-promoting effects, such as those of microorganisms inoculated with the compost, are postulated.

### 3.3. Endosphere and Rhizosphere Prokaryotic Communities

The endosphere prokaryotic communities in carrot roots are shown in Figures S1–S3 at the phylum, family, and genus levels, respectively. Similarly, the carrot rhizosphere prokaryotic communities are shown in Figures S4–S6. Most of them were bacteria, but a few archaea were also detected. At the phylum level, Proteobacteria and Firmicutes were dominant in the endosphere, while the rhizosphere samples were dominated by Proteobacteria, Crenarchaeota, Actinobacteria, and Firmicutes. At the family level, Bacillales, Pedosphaeraceae, Rhizobiaceae, Oxalobacteraceae, and Spingobacteriaceae were dominant in the endosphere samples. The genus-level composition of the endosphere samples included unclassified Microscillaceae, unclassified Sandaracinaceae, *Sphingomonas*, *Uliginosibacterium*, *Asticcacaulis*, and *Acidibacter*. On the other hand, the rhizosphere samples were dominated by *Bacillus*, Candidatus_Nitrosotalea, unclassified Nitrososphaeraceae, *Geobacillus*, unclassified Vicinamibacterales, unclassified Gemmatimonadaceae, and unclassified Bacillales.

**Figure 2:**
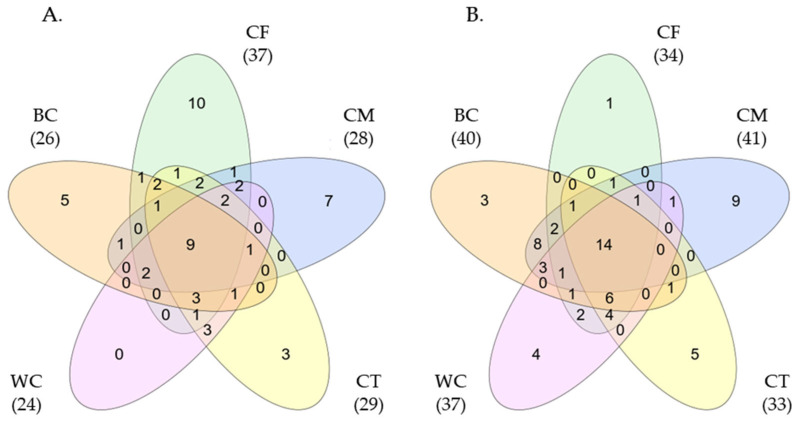
Venn diagram showing the number of unique and shared endosphere prokaryotic taxa at the genus level among the treatments at 60 DAS (**A**) and 108 DAS (**B**). Each of the colored ovals represents a treatment. Values within the intersections represent the shared genera, while values outside the intersections are unique to each compartment. The total number of taxa detected in each treatment is shown in parentheses. The meanings of the abbreviations of the treatments are given in Table 2.

The Venn diagram analysis identified 58 and 68 endosphere prokaryotic genera at 60 and 108 DAS, respectively, when all treatments were grouped together (Figure 2). At 60 DAS, a subset of 18 (31.0% in total) prokaryotic taxa were shared by four or more treatments, while 27 taxa (46.6%) were shared by two or fewer treatments. Similarly, at 108 DAS, 23 (39.7% in total) taxa were commonly found in four or more treatments, but 34 taxa (58.6%) were detected specifically in one or two treatments.

*Burkholderia*-*Caballeronia*-*Paraburkholderia*, as well as *Rhodanobacter*, Entomoplasmatales_type_III, *Acidibacter*, *Dyella*, *Citrifermentans*, uncultured Rhodocyclaceae, and *Clostridium*_sensu_stricto_10, were detected in the endosphere of all treatments at both 60 DAS and 108 DAS. *Allorhizobium*-*Neorhizobium*-*Pararhizobium*-*Rhizobium*, *Bradyrhizobium*, *Sphingomonas*, *Bordetella*, and Unassigned Comamonadaceae were further detected in the endosphere of all treatments at 108 DAS. The genera *Burkholderia*, *Sphingomonas*, and *Rhodanobacter* have been reported to contain strains exhibiting plant growth-promoting and biocontrol activities [30,31,32]. The results of this study suggest that these beneficial bacteria are contained as core endophytes in carrot roots and play an important ecological role.

The richness and diversity of the prokaryotic community are shown in Table 5. The fertilizers did not affect the richness, evenness, and Shannon indexes in the rhizosphere and endosphere prokaryotic communities (Tukey’s test, *p* > 0.05). However, in terms of Faith pyrogenetic diversity (PD), the CM and BC treatments were significantly higher than those of the control (CT). According to the results of two-way ANOVA, the differences in the treatment affect the richness and Faith PD of the endosphere prokaryotic communities, and the differences in the sampling time affect all indicators of the endosphere prokaryotic communities (Table 6). However, no significant effects were found on the rhizosphere prokaryotic communities, indicating that the effects are specific to the endosphere.

The richness and diversity in the endosphere were clearly lower than those in the rhizosphere. This is a known phenomenon in other crops: for example, Ahmed et al. [33] showed that higher microbial diversity existed in the rhizosphere soils of tobacco (*Nicotiana tabacum*) than in the roots, and Zhang et al. [34] reported that the diversity and network complexity of the rice-associated microbiome decreased steadily from far to near the roots, from the rice exterior to interior, and from belowground to aboveground niches.

**Figure 3:**
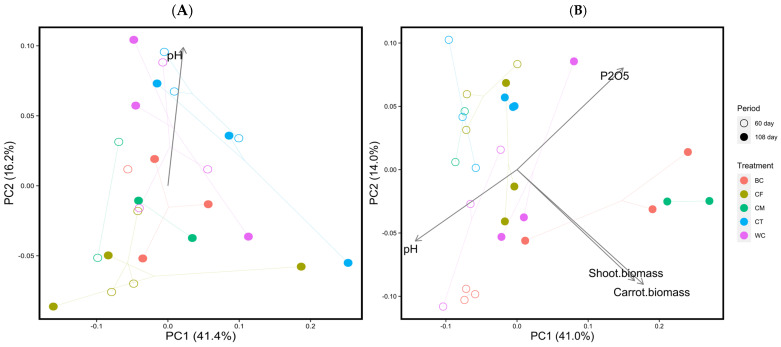
Results of the PCoA analysis of soil prokaryotic communities based on weighted UniFrac for the 60 and 108 DAS samples: rhizosphere soil (**A**) and endosphere soil (**B**). “day” means the day after sowing [DAS]. Arrows represent significantly correlated environmental and carrot growth factors (*p* < 0.05). The meanings of the abbreviations of the treatments are given in Table 2.

The results of the PCoA analysis of the soil prokaryotic communities based on weighted UniFrac for the 60 and 108 DAS showed a larger temporal shift of the endosphere prokaryotic community in the BC and CM treatments than the others (Figure 3). However, there were no clear differences among the treatments in the rhizosphere prokaryotic community. This was also supported by the results of PERMANOVA for weighted UniFrac distances of rhizosphere and endosphere prokaryotic communities (Table 7).

**Figure 4:**
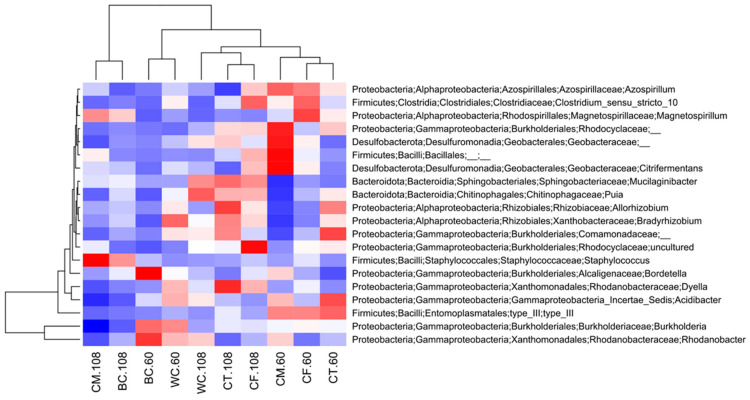
Heatmap and hierarchical clustering of relative abundance of top 20 prokaryotic genera detected in the carrot endosphere. The figures after the treatment names, “60” and “108”, mean 60 and 108 DAS, respectively. The meanings of the abbreviations of the treatments are given in Table 2. *Burkholderia* and *Allorhizobium* refer to *Burkholderia*-*Caballeronia*-*Paraburkholderia* and *Allorhizobium*-*Neorhizobium*-*Pararhizobium*-*Rhizobium* in the SILVA 138 database, respectively.

The heatmap and hierarchical clustering of the relative abundance of the top 20 prokaryotic genera detected in the carrot endosphere are shown in Figure 4. This figure also indicates that the endosphere prokaryotic communities at 108 DAS in the BC and CM treatments were different from the others.

Endophytes have the potential to help carrot plants withstand a wide range of biotic and abiotic stresses, resulting in improved crop performance and reduced reliance on agrochemicals to address production challenges [23]. In this study, we observed that *Bradyrhizobium*, *Streptomyces*, *Pseudomonas*, *Massilla*, Microscillaceae, *Sphingomonas*, and *Acidibacter* were the dominant members of root endophytes, which is consistent with previous studies [21,23]. Considering that endophytes must possess specific characteristics such as high motility and extracellular polymeric substance production to colonize roots, it is not surprising that a selective group of taxa often consistently represents the majority of endophytes. Most endophytic bacteria, including *Pseudomonas* and *Streptomyces*, play important roles in carrot development, as Rodriguez et al. [35] showed when demonstrating that endophytic microorganisms may be involved in the enantioselective reduction in ketones and ketoesters in fresh carrot root pieces. Therefore, it is important to understand the factors that influence carrot endophytic communities.

In other crops, there is evidence that organic matter application affects endophytic communities. For example, Hallmann et al. [36] found an increased abundance of the bacterium *Burkholderia cepacia* in cotton roots grown in soil amended with organic matters, while roots grown in unamended soil had very little colonization. Some studies have shown greater potential for disease-suppressive activity in organic farming systems compared with conventional farming systems, which is thought to be related to changes in the soil’s microbial community composition induced by organic matter application [37]. A previous study showed that soils amended with organic matters had greater microbial biomass and activity than the soils in the conventional system, and that the composition of endophytes in carrot roots was more abundant and diverse and had greater antagonistic activity [23]. Our results showing that organic matter application altered the composition of the endophytic bacterial community in carrot roots agree with previous studies.

Under natural conditions, plants are always associated with a well-organized microbe community, which is essential for plant growth and function, and the association is termed holobiont [38]. The same is true for crops, and the holobiont-constituting microorganisms support the host plant through plant-growth-promoting activities such as IAA production, N fixation, and disease resistance. For example, it has been reported that healthy tobacco plants exhibited high microbial diversity compared with diseased plants, and bacterial genera such as *Bacillus*, *Bradyrhizobium*, *Ensifer*, *Neorhizobium*, and *Lysobacter* that are related to plant growth promotion and disease-suppressing abilities were more dominant in the roots and rhizosphere soil than potentially pathogenic fungal genera [33]. It is also known that some bacterial isolates [39] or probiotic communities [40] can change the diversity and composition of rhizosphere microbial communities and support plant health. In the case of Sanchi ginseng (*Panax notoginseng*), a Chinese medicinal plant belonging to the same order as carrot (order Apiales), whose roots are used by humans, an active probiotic community was developed to reshape the soil microbiota and restrain root-rot disease caused by *Fusarium oxysporum* [40]. To achieve adequate productivity of carrots without excessive reliance on chemical fertilizers and pesticides, it is desirable to establish technologies for controlling the entire prokaryotic community in the rhizosphere and endosphere for healthy growth.

## 4. Conclusions

In this study, the impact of organic matter application on bacterial and archaeal community formation in the carrot rhizosphere and endosphere was investigated. As a result, we found that bacterial and archaeal communities in the carrot rhizosphere showed little temporal shift in terms of carrot growth and little effect of organic matter application. In contrast, the endosphere prokaryotic communities showed a greater temporal shift and the effect of the type of organic matter applied also appeared here. In this study, the genera such as *Burkholderia*, *Sphingomonas*, *Rhodanobacter* were detected as the dominant ones regardless of the treatments. Since they exhibit plant-growth-promoting and biocontrol activities, they may play an important ecological role as the core endophytes in carrot roots. It is necessary to clarify why different types of organic matter have different effects on prokaryotic communities in the roots. Although only prokaryotic communities were analyzed in this study, the effects on fungal communities in the rhizosphere and endosphere should also be investigated. In addition, the growth of carrots was most favorable when bark compost was applied, suggesting that the microorganisms inoculated with this compost may have had a growth-promoting effect. In the future, it will be necessary to isolate useful microorganisms and examine their potential uses in agriculture.

## Figures and Tables

**Table 1:** The target appreciation dose of fertilizer in this study.

Component	N	P_2_O_5_	K_2_O
Target dose	200 kg-N ha^−1^	50 kg-P_2_O_5_ ha^−1^ (22 kg-P ha^−1^)	120 kg-K_2_O ha^−1^ (100 kg-K ha^−1^)

**Table 2:** Fertilizer treatments examined in this study.

Treatment	Fertilizer ^1^	Amount
Control (CT)	None	None
Chemical Fertilizer (CF)	AS	16.9 g planter^−1^
	FMP	5.1 g planter^−1^
	KCl	3.3 g planter^−1^
Cattle Manure (CM)	CM	79.0 g planter^−1^ (4.55 Mg ha^−1^)
	AS	12.7 g planter^−1^
	KCl	3.3 g planter^−1^
Bark Compost (BC)	BC	124 g planter^−1^ (7.14 Mg ha^−1^)
	AS	12.7 g planter^−1^
	KCl	2.5 g planter^−1^
Weed Compost (WC)	WC AS	174 g planter^−1^ (10 Mg ha^−1^) 6.8 g planter^−1^

^1^ As: ammonium sulfate, FMP: fused magnesium phosphate, KCl: potassium chloride, CM: cattle manure, BC: bark compost, and WC: weed compost.

**Table 3:** Changes in soil physicochemical properties ^¶^.

	pH		EC (μS/cm)		Available P (g-P_2_O_5_/kg-ds)	
Treatment	60 DAS	108 DAS	60 DAS	108 DAS	60 DAS	108 DAS
CT	4.33 ± 0.04 ab	4.25 ± 0.04 a	0.57 ± 0.20 a	0.29 ± 0.14 a	0.30 ± 0.04 b	0.48 ± 0.02 b
CF	4.21 ± 0.01 c	4.13 ± 0.04 b	0.72 ± 0.31 a	0.36 ± 0.08 a	0.26 ± 0.02 ab	0.42 ± 0.00 a
CM	4.18 c	4.11 b	0.85 a	0.48 a	0.24 ab	0.42 ab
BC	4.40 ± 0.07 a	4.14 ± 0.03 b	0.97 ± 0.37 a	0.59 ± 0.21 a	0.26 ± 0.01 ab	0.40 ± 0.02 a
WC	4.25 ± 0.03 bc	4.21 ± 0.05 ab	0.47 ± 0.22 a	0.39 ± 0.18 a	0.22 ± 0.03 a	0.41 ± 0.02 a
			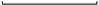 *		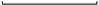 ***	
	**TC (mg-C/g-ds)**		**TN (mg-N/g-ds)**		**C/N ratio**	
**Treatment**	**60 DAS**	**108 DAS**	**60 DAS**	**108 DAS**	**60 DAS**	**108 DAS**
CT	12.2 ± 0.1 a	12.0 ± 0.2 a	1.2 ± 0.1 a	1.1 ± 0.0 a	10.1 ± 0.7 a	11.4 ± 0.3 a
CF	12.4 ± 0.1 a	12.3 ± 0.6 a	1.2 ± 0.1 a	1.1 ± 0.0 a	10.4 ± 0.7 a	10.8 ± 0.2 a
CM	12.6 a	12.4 a	1.2 a	1.2 a	10.3 a	10.8 a
BC	13.0 ± 0.1 a	12.7 ± 1.4 a	1.3 ± 0.1 a	1.2 ± 0.3 a	10.3 ± 0.7 a	10.7 ± 1.0 a
WC	12.6 ± 0.1 a	12.4 ± 0.4 a	1.1 ± 0.1 a	1.2 ± 0.0 a	11.0 ± 0.6 a	10.6 ± 0.6 a
	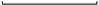 *					

^¶^ mean ± 1 standard deviation (*n* = 3) is presented except for CM, where only means are shown because of lacking data, and “ds” means “dry soil”. Different letters indicate significant differences between the treatments (Tukey’s test, *p* < 0.05). * and *** mean significant differences between the sampling dates (paired *t*-test, *p* < 0.05, and 0.001, respectively). The meanings of the abbreviations of the treatments are given in Table 2.

**Table 4:** The effect of organic and chemical fertilizers on carrot growth ^¶^.

	Shoot Biomass (g-dw/Plant)		Taproot Biomass (g-dw/Plant)	
Treatment	60 DAS	108 DAS	60 DAS	108 DAS
CT	0.62 ± 0.68 a	1.69 ± 0.96 a	0.18 ± 0.25 a	2.77 ± 1.85 a
CF	0.70 ± 0.38 a	2.33 ± 0.53 a	0.50 ± 0.26 ab	4.08 ± 1.14 a
CM	0.93 a	2.68 a	0.60 ab	3.84 a
BC	1.64 ± 0.46 a	2.90 ± 0.15 a	1.86 ± 1.00 b	7.66 ± 0.74 b
WC	0.46 ± 0.19 a	1.43 ± 0.79 a	0.23 ± 0.08 a	2.50 ± 1.26 a
	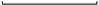 **		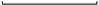 *	

^¶^ means ± standard deviation (*n* = 3) is presented, except for CM where only means are shown because of lacking data, and “dw” means “dry weight”. Different letters indicate significant differences between the treatments (Tukey’s test, *p* < 0.05). * and ** mean significant differences between the sampling dates (paired *t*-test, *p* < 0.05, and 0.01, respectively). The meanings of the abbreviations of the treatments are given in Table 2.

**Table 5:** Richness and diversity of the prokaryotic community ^¶^.

	Period	Treatment	Richness	Shannon	Faith PD	Evenness
Rhizosphere	60 DAS	CT	542 ± 74	8.35 ± 0.23	44.9 ± 3.5	0.92 ± 0.00
		CF	526 ± 39	7.84 ± 0.30	40.3 ± 0.9	0.87 ± 0.02
		CM	541 ± 57	8.08 ± 0.31	44.5 ± 4.2	0.89 ± 0.02
		BC *	442	7.97	38.5	0.91
		WC	640 ± 25	8.48 ± 0.06	48.0 ± 0.9	0.91 ± 0.01
	108 DAS	CT	477 ± 38	7.82 ± 0.37	39.9 ± 1.9 c	0.88 ± 0.04
		CF	458 ± 67	7.35 ± 0.46	38.8 ± 4.2 bc	0.83 ± 0.03
		CM	538 ± 30	8.20 ± 0.04	42.1 ± 1.7 a	0.90 ± 0.00
		BC	574 ± 40	8.19 ± 0.09	43.8 ± 2.3 ab	0.89 ± 0.01
		WC	539 ± 42	8.29 ± 0.20	43.5 ± 2.4 abc	0.91 ± 0.01
Endosphere	60 DAS	CT	137 ± 25	5.75 ± 0.04	15.2 ± 2.1	0.82 ± 0.03
		CF	113 ± 12	5.80 ± 0.08	12.9 ± 0.3	0.85 ± 0.01
		CM	86 ± 14	5.27 ± 0.07	11.6 ± 1.5	0.83 ± 0.04
		BC	147 ± 15	4.88 ± 0.19	16.4 ± 1.7	0.68 ± 0.04
		WC	139 ± 21	5.11 ± 0.61	13.9 ± 1.5	0.72 ± 0.07
	108 DAS	CT	172 ± 33	6.13 ± 0.15	17.6 ± 3.0	0.83 ± 0.01
		CF	181 ± 8	6.14 ± 0.16	18.4 ± 0.8	0.82 ± 0.02
		CM	376 ± 75	7.69 ± 0.20	37.6 ± 4.0	0.90 ± 0.01
		BC	365 ± 77	7.28 ± 0.62	33.8 ± 5.9	0.86 ± 0.04
		WC	203 ± 12	6.29 ± 0.23	21.4 ± 1.1	0.82 ± 0.03

^¶^ Different letters indicate significant differences (Tukey’s test, *p* < 0.05). * This sample has one replication due to the lack of sequencing depth of other replicants. The meanings of the abbreviations of the treatments are given in Table 2.

**Table 6:** Results of two-way ANOVA on richness and alpha diversities of prokaryotic communities.

	Rhizosphere				Endosphere			
Factor	Richness	Shannon	Faith	Evenness	Richness	Shannon	Faith	Evenness
A	F4,16 = 1.20	F4,16 = 2.28	F4,16 = 1.46	F4,16 = 2.62	F4,18 = 3.78 *	F4,18 = 1.24	F4,18 = 5.59 **	F4,18 = 2.36
B	F1,16 = 1.51	F1,16 = 1.75	F1,16 = 1.77	F1,16 = 1.40	F1,18 = 30.40 ***	F1,18 = 36.66 ***	F1,18 = 39.13 ***	F1,18 = 8.42 **
A × B	F4,16 = 1.14	F4,16 = 0.59	F4,16 = 0.78	F4,16 = 0.60	F4,18 = 4.43 *	F4,18 = 4.63 **	F4,18 = 5.56 **	F4,18 = 2.60

A: Treatment, B: Time. Significantly different at *** *p* < 0.001, ** *p* < 0.01, and * *p* < 0.05.

**Table 7:** Permutational multivariate analysis of variance (PERMANOVA) table for weighted UniFrac distances of rhizosphere and endosphere prokaryotic communities with 9999 permutations.

	Rhizosphere				Endosphere			
Factor	Df	Sum of Sqs	F	R2	Df	Sum of Sqs	F	R2
Treatment (A)	4	0.13	1.68 *	0.256	4	0.16	2.59 **	0.228
Time (B)	1	0.02	1.26	0.048	1	0.14	8.86 ***	0.195
A × B	4	0.04	0.57	0.087	4	0.13	2.08 *	0.183
Residual	16	0.30		0.609	18	0.28		0.395
Total	25	0.50		1.000	27	0.71		1.000

Significantly different at *** *p* < 0.001, ** *p* < 0.01, and * *p* < 0.05.

## Data Availability

The data presented in this study are openly available in the DDBJ Sequenced Read Archive under the accession number DRA017099.

## Supplementary Materials

The following supporting information can be downloaded at: https://www.mdpi.com/article/10.3390/microorganisms11102377/s1, Table S1: Components of N, P_2_O_5_, and K_2_O in the fertilizers used in this study (mg g^−1^); Figure S1: Top 10 phyla of endosphere prokaryotes in carrot roots (“day” means the day after sowing [DAS]); Figure S2: Top 20 families of endosphere prokaryotes in carrot roots (“day” means the day after sowing [DAS]); Figure S3: Top 20 genera of endosphere prokaryotes in carrot roots (“day” means the day after sowing [DAS]); Figure S4: Top 10 phyla of rhizosphere prokaryotes in carrot roots (“day” means the day after sowing [DAS]); Figure S5: Top 20 families of rhizosphere prokaryotes in carrot roots (“day” means the day after sowing [DAS]); and Figure S6: Top 20 genera of rhizosphere prokaryotes in carrot roots (“day” means the day after sowing [DAS]).
