# Supplementary material for: The Application of Organic Matter Temporarily Shifts Carrot Prokaryotic Communities in the Endosphere but Not in the Rhizosphere
Source: Microorganisms. 2023 Sep 23;11(10):2377. doi: 10.3390/microorganisms11102377 (PMC10608867; doi:10.3390/microorganisms11102377)
Supplement: Supplementary file 1 [file microorganisms-11-02377-s001.zip › microorganisms-2615485-supplementary.pdf]

Supplemental materials

The Application of Organic Matter Temporarily Shifts Carrot Prokaryotic Communities in the Endosphere but Not in the Rhizosphere

Irem Bagci [1], Kazuki Suzuki [2], Rasit Asiloglu [2], and Naoki Harada [2,*]

[1] Graduate School of Science and Technology, Niigata University, Niigata 950-2181, Japan; irembagci98@gmail.com

[2] Institute of Science and Technology, Niigata University, Niigata 950-2181, Japan; suzukik@agr.niigata-u.ac.jp (K.S.); asiloglu@agr.niigata-u.ac.jp (R.A.)

[*] Correspondence: naharada@agr.niigata-u.ac.jp

Table S1. Components of N, P, and K in the fertilizers used in this study.

Name (abbreviation)	Ammonium sulfate (AS)	Fused Magnesium Phosphate (FMP)	Potassium chloride (KCl)	Cattle manure (CM)	Bark compost (BC)	Weed compost (WC)
N: mg-N g^{-1}	20.5	0	0	1.1	0.7	1.2
P: mg-P_2O_5 g^{-1}	0	17	0	1.1	0.7	0.5
K: mg-K_2O g^{-1}	0	0	63.2	1.7	0.4	1.2

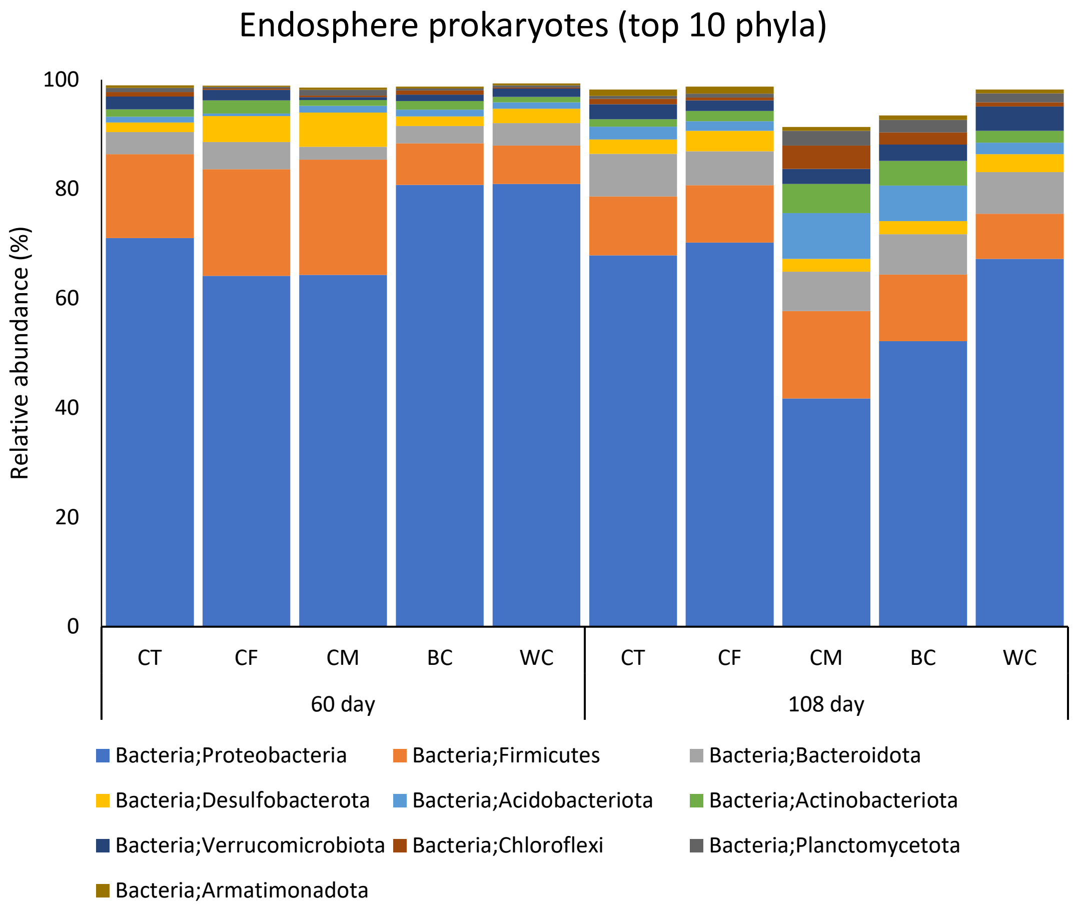

Figure S1. Top 10 phyla of endosphere prokaryotes in carrot roots ("day" means the day after sowing [DAS])

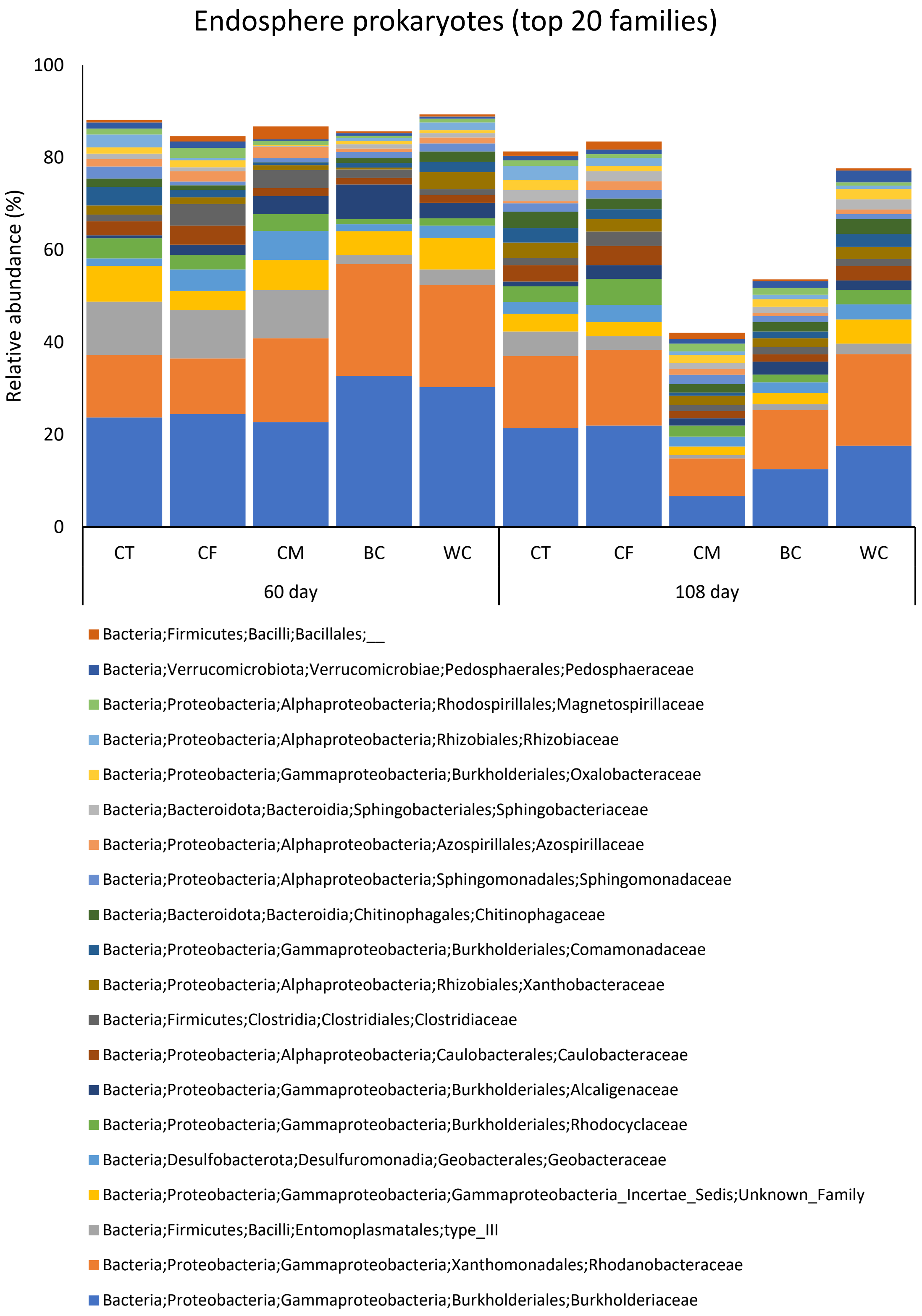

Figure S2. Top 20 families of endosphere prokaryotes in carrot roots ("day" means the day after sowing [DAS])

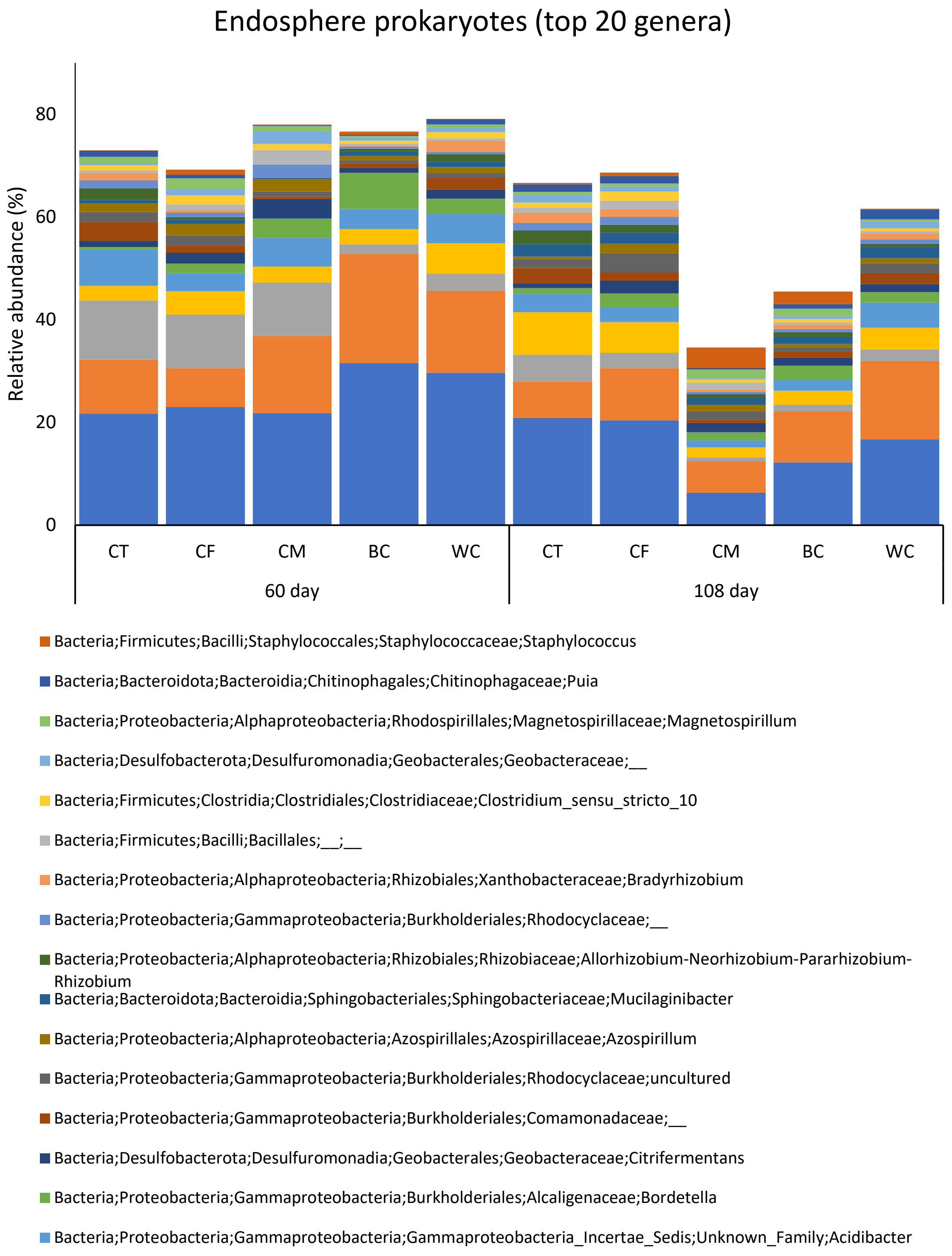

Figure S3. Top 20 genera of endosphere prokaryotes in carrot roots ("day" means the day after sowing [DAS]). *Burkholderia* and *Allorhizobium* refer to *Burkholderia-Caballeronia-Paraburkholderia* and *Allorhizobium-Neorhizobium-Pararhizobium-Rhizobium* in the SILVA 138 database respectively.

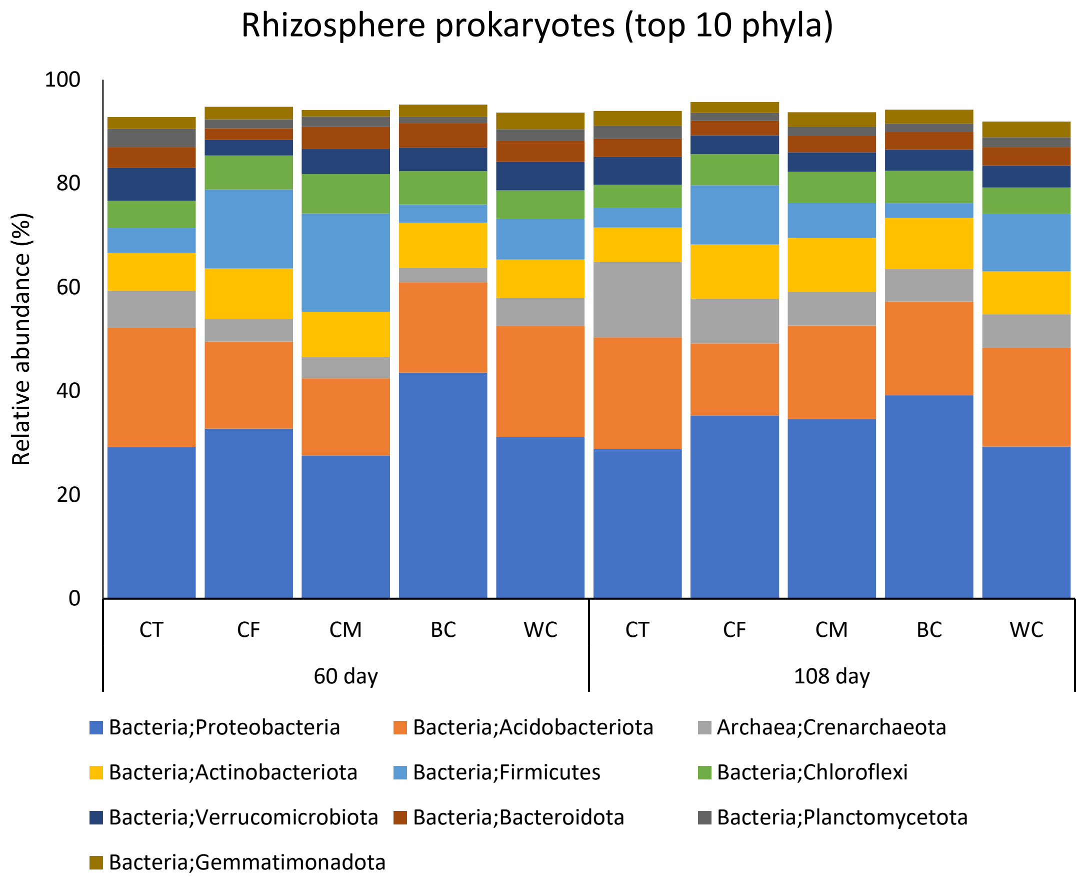

Figure S4. Top 10 phyla of rhizosphere prokaryotes in carrot roots ("day" means the day after sowing [DAS])

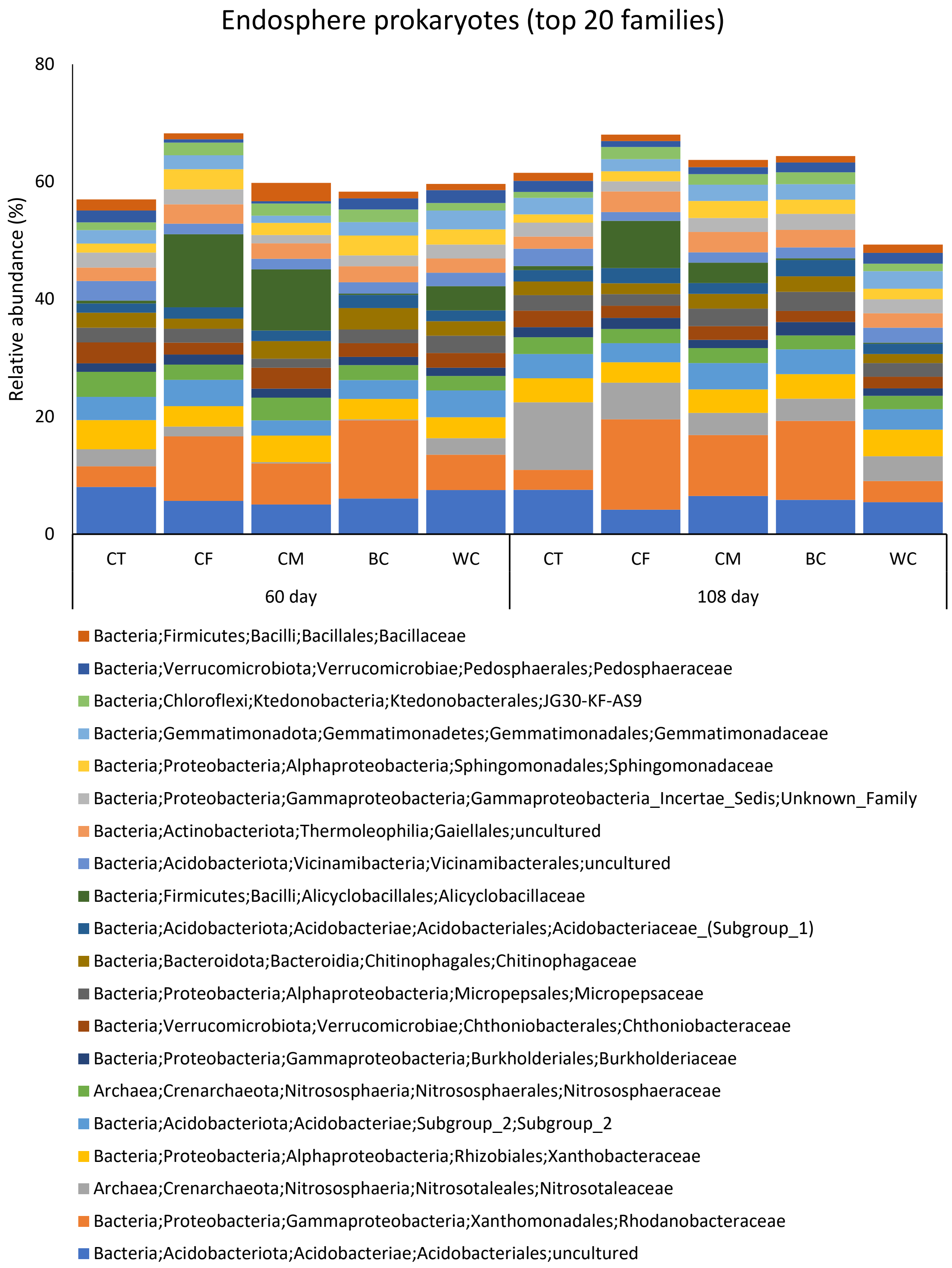

Figure S5. Top 20 families of rhizosphere prokaryotes in carrot roots ("day" means the day after sowing [DAS])

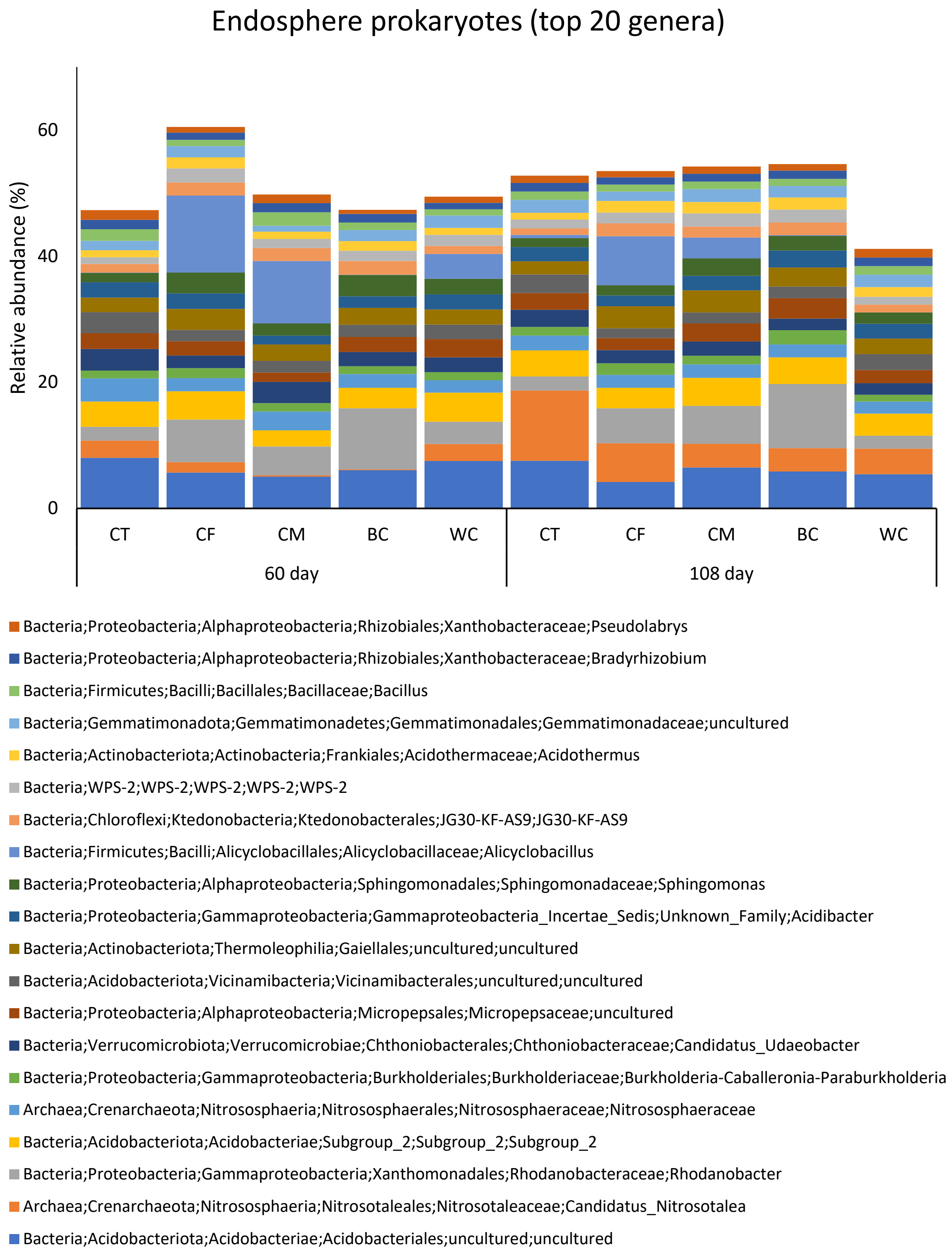

Figure S6. Top 20 genera of rhizosphere prokaryotes in carrot roots ("day" means the day after sowing [DAS]). *Burkholderia* and *Allorhizobium* refer to *Burkholderia-Caballeronia-Paraburkholderia* and *Allorhizobium-Neorhizobium-Pararhizobium-Rhizobium* in the SILVA 138 database respectively.
